# A systematic review of CXCL13 as a biomarker of disease and treatment response in rheumatoid arthritis

**DOI:** 10.1186/s41927-020-00154-3

**Published:** 2020-11-02

**Authors:** Katie Bechman, Anthony Dalrymple, Charles Southey-Bassols, Andrew P. Cope, James B. Galloway

**Affiliations:** Centre of Rheumatic Diseases, Weston Education Centre, King’s College London, Room 3.46, Third Floor, London, SE5 9RJ UK

## Abstract

**Background:**

The B cell chemoattractant CXCL13 is a promising biomarker in rheumatoid arthritis (RA), with a plausible role in supporting diagnosis, monitoring disease activity and as a prognostic value. It is a key chemokine driving the formation of lymphoid follicles within the inflamed synovium. The objective of this systematic review was to evaluate the role of CXCL13 as a viable biomarker in RA.

**Methods:**

We conducted a systematic literature review of all published cohort and randomised controlled trials evaluating the role of CXCL13 in RA. The primary outcomes were; i) CXCL13 levels in RA patients compared to healthy controls, ii) the correlation between CXCL13 and markers of disease activity, and iii) the association between CXCL13 and treatment response.

**Results:**

The search produced 278 articles, of which 31 met the inclusion criteria. Of the 12 studies evaluating CXCL13 expression in early or established RA, all reported higher levels than that seen in healthy controls. Twelve of sixteen studies reported a weakly positive correlation between CXCL13 and markers of disease activity including DAS28 and swollen joint count, with rho values between 0.20–0.67. In 2 studies, CXCL13 levels correlated with ultrasonographic evidence of synovitis. Eighteen studies assessed CXCL13 in response to therapeutic intervention. The majority signified a fall in levels in response to treatment including biologics and Janus kinase (JAK) inhibition. In some, this reduction was only seen in treatment responders. High CXCL13 levels predicted failure to achieve disease remission with csDMARDs. The evidence for treatment prediction with biologics was conflicting.

**Conclusion:**

Despite evidence to suggest a role in diagnosing RA and in detecting synovitis, the heterogeneity of studies included in this review limit our ability to draw robust conclusions. At present there are inadequate results to justify the routine use of CXCL13 as a biomarker in RA routine clinical practice.

## Background

Rheumatoid arthritis (RA) is a chronic, systemic immune-mediated disease characterised by synovial inflammation and progressive joint destruction [1]. Although the classical paradigm of RA pathogenesis is centred on the role of the CD4+ T cells, it is clear that B cells also demonstrate a vital role including cytokine secretion, antigen presentation, and interaction with other inflammatory cells [2, 3]. Autoreactive B cells are also activated by T cells to produce IgG autoantibodies that may be directly involved in joint damage [2, 3].

Existing biomarkers in RA, including acute phase reactants have their limitations. C-reactive protein (CRP) is a sensitive measure of inflammation but lacks disease specificity for joint inflammation and elevated levels are seen in intercurrent illness or with obesity [4]. The erythrocyte sedimentation rate (ESR) is non-specific, influenced by confounding factors such as age, sex, anaemia, renal disease, immunoglobulin and fibrinogen levels [5]. It is not sensitive to short-term changes, with a slow response to the acute phase reaction leading to false negatives in an early inflammatory process [5]. Both CRP and ESR lack sensitivity in patients with limited small joint or predominantly lower limb involvement and in those receiving immunosuppression with steroids or drugs that target interleukin-6 (IL-6) e.g. tocilizumab [6]. IL-6 is a key driver of the acute phase response with an important role in the liver’s production of CRP. Rheumatoid factor (RF) and anti-citrullinated peptide (ACPA) have value in diagnostics, although may be negative in up to 30% of patients [7, 8]. They do have use as a prognostic marker of disease severity but not as markers of disease activity [9]. There is a current need for a biomarker with improved sensitivity which can aid diagnosis, monitor disease activity and provide prognostic value.

CXCL13, also known as B-lymphocyte chemoattractant (BLC) or C-X-C motif chemokine 13, is a key chemokine involved in the positioning and activation of cells at lymphoid and extra-lymphoid sites. It binds to its cognate receptor CXCR5, and attracts CXCR5-expressing B cells and follicular T-helper cells [10]. Overexpression of CXCL13 in extra-lymphoid structures enhances B cell migration and promotes ectopic lymphoid neogenesis [11]. In the RA inflammatory responses, TNF-alpha and IL-6 upregulate CXCL13 expression, driving the development of B cell follicles and germinal centre reactions within the synovium [10, 12]. The CXCL13-CXCR5 interaction enables B-cell maturation into plasma cells and the production of antibodies [13].

CXCL13 is of interest as a biomarker in RA, with a profound effect on shaping local synovial architecture and may provide a signature of a specific RA disease subset. Several studies have investigated the link between CXCL13, disease activity, serological markers (such as RF and ACPA) and change in response to treatment. To our knowledge, these studies have not been analysed collectively. Our objective was to evaluate the current existing literature in RA, to assess how CXCL13 correlates with disease activity, treatment response, and to determine its use as a potential biomarker in clinical practice.

## Methods

### Search strategy

The literature was searched systematically by two investigators (CS and AD) using MEDLINE and EMBASE databases. The search terms used were ‘rheumatoid arthritis’ OR ‘inflammatory arthritis’ AND ‘CXCL13’ OR ‘Chemokine Ligand 13’. The search was undertaken in February 2018 and re-run prior to the final analysis to identify new studies that could be retrieved for incorporation in the systematic review.

### Study selection

We identified English language publications of cohort or randomised controlled trials (RCTs). Publications were included if they met the following eligibility criteria: 1) the study evaluated CXCL13 levels 2); the study included patients diagnosed with RA based on the American College of Rheumatology (ACR) criteria. Studies presenting duplicate data were excluded. Titles and abstracts were screened independently by two investigators (CS and AD). The full text of the potential studies for inclusion were retrieved and assessed for eligibility. Data was extracted independently (by CS and AD). Disagreements over study eligibility were resolved through discussion with a third reviewer (KB). The primary outcome of interest was i) the level of CXCL13 in RA patients compared to healthy control (HC) and ii) the correlation between CXCL13 levels and markers of disease activity and iii) the association between CXCL13 and treatment response.

## Results

**Fig. 1:**
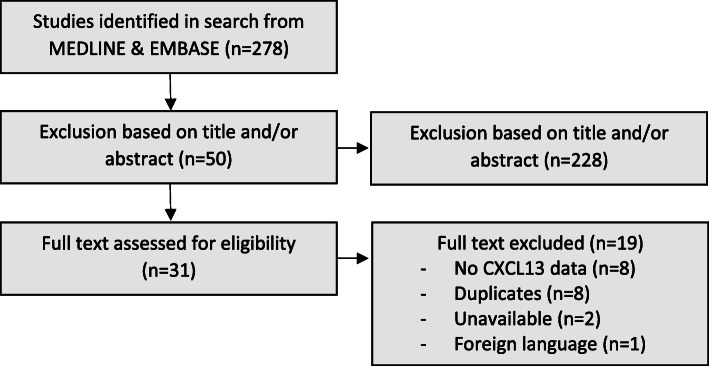
Flow chart of studies included in the systematic review

The search identified 278 articles. Of these, 228 were excluded based on title and abstract. A further 19 studies were excluded after full paper review. Figure 1 summarises the flowchart in accordance with the PRISMA statement [9]. In total, 31 studies were included, comprising 20 published articles and 11 conference abstracts. Of these studies, 8 were randomised controlled trials (RCTs) and 23 observational cohorts (Table 1). The total number of patients analysed were 5894, with a median population of 132 (IQR: 57–242) in the RCTs and 147 (IQR: 30–225) in the observational cohorts. Nearly all RCTs evaluated CXCL13 levels in patients with established RA (EstRA), whilst observational cohorts assessed patients with both early (ERA) and established disease.

**Table 1:** CXCL13 in RA versus healthy controls and correlation with disease activity

Study	Population	Substrate	RA vs healthy controls	CXCL13 level (pg/ml)	Correlation of CXCL13 with disease activity
Pandya et al. (2017) [14]	*N* = 57. ERA. preDMARD	Serum by ELISA	ERA > HC	Not published	No correlation (data not presented): DAS28-CRP, DAS28-ESR, ESR, SJC28
Moura et al. (2017) [15]	*N* = 33. ERA, EstRA csDMARD, DMARD	Serum by ELISA	ERA/EstRA > HC ERA = EstRA	Not published (individual levels in figure)	No correlation (data not presented): DAS28-ESR, ESR, SJC28, TJC28
Han et al. (2016) [16]	*N* = 29. EstRA TNFi (ADA & ETN)	Serum by ELISA		RA RF+: 372 (± 649), RA RF-: 40 (± 56)	No correlation (data not presented): DAS28-ESR, ESR but correlated with change in DAS28-ESR (*r* = 0.54). Higher in ACPA & RF positive patients
Kumagai et al.* (2016) [17]	*N* = 146. EstRA bDMARD	Serum by ELISA	EstRA > HC	Not published	Correlation: DAS28-CRP (*r* = 0.52)
Loza et al.* (2016) [18]	*N* = 916. ERA Anti-IL6	Serum by ELISA/MSD /Luminex	EstRA > HC	Not published	
Bugatti et al.* (2016) [19]	*N* = 213. EstRA csDMARD	Both, method not published		Not published	Lower correlation with disease activity compared to CRP (data not presented). Predict failure to achieve LDA. Correlated with RF (data not presented). Higher in ACPA+ patients.
Han et al. (2015) [20]	*N* = 111. ERA csDMARD, DMARD	Serum by ELISA	EstRA > HC	Not published	
Yeo et al.* (2015) [21]	*N* = 29. ERA preDMARD	Synovial by mRNA qPCR		Not published	Correlation: US power doppler (data not presented)
Bugatti et al.* (2014) [22]	*N* = 71. ERA, EstRA csDMARD	Synovial by mRNA qPCR		Not published	ACPA & radiographic erosive disease associated greater expression CXCL13 (data not presented)
Greisen et al. (2014) [23]	*N* = 114. ERA csDMARD, TNFi	Serum by ELISA	ERA > HC	RA: 149 (75–245) HC: 50 (29–93)	Correlation: B/L SJC28 (*r* = 0.34) PGA (*r* = 0.38) not TJC28
Bugatti et al.* (2014) [24]	*N* = 205 csDMARD	Serum by ELISA		Not published	Correlation: ESR (*r* = 0.35), CRP (*r* = 0.36) DAS28 & US grey scale/power doppler (data not presented)
Moura et al. (2014) [25]	Unpublished	Serum by ELISA	ERA/EstRA > HC	Not published	No correlation: DAS28-ESR, ESR, SJC28, TJC28 (data not presented)
Jones et al. (2014) [26].	EstRA *N* = 193 ERA *N* = 339	Serum by ELISA		EstRA RF+: 331 (250–431), EstRA RF-: 93 (71–124), ERA RF+: 324 (224–478), ERA RF-: 50 (35–78)	Correlation DAS28-CRP (r = 0.52) in EstRA. Correlation RF in ERA (r = 0.54) & EstRA (0.45)
Sellam et al. (2013) [27]	*N* = 278. ERA	Serum by ELISA	ERA > HC		Correlation: DAS28-CRP (*r* = 0.20)
Sherif et al. (2013) [28]	*N* = 30. ERA	Serum by ELISA	ERA > HC	RA + sSS: 137(±63), RA -sSS: 165(±91), HC: 12 (±2.2)	Correlation: DAS28-ESR (*r* = 0.68), disease duration (*r* = 0.41)
Ahmed et al. (2013) [29]	*N* = 30. ERA	Serum by ELISA	ERA > HC	RA: 120–350 HC: 8–30	Correlation: DAS28-ESR (0.42) US power doppler (*r* = 0.69)
Setiadi et al.* (2013) [30]	*N* = 1135. ERA	Serum, method not published	ERA > HC	Not published	
Bugatti et al. (2012) [31]	*N* = 180. ERA	Serum by ELISA	ERA > HC	RA: 73 (47–117), HC: 54 (42–63)	Correlation: DAS44-ESR (*r* = 0.35) SJC44 (*r* = 0.28) CRP (*r* = 0.42) ESR (*r* = 0.41) US grey scale (*r* = 0.27) US power doppler (*r* = 0.26)
Meeuwisee et al. (2011) [32]	*N* = 229. ERA	Serum by ELISA		Cohort 1: 167 (±86), Cohort 2: 156 (±99)	Correlation: CRP (*r* = 0.43), ESR (*r* = 0.30), SJC66 (*r* = 0.23). Associated with radiographic erosive disease and failure to achieve remission. Levels higher in RF/ACPA patients.

Unclear whether the two publications by Moura et al., in abstract form from 2014 and full publication from 2017 involve an analysis of the same patient population

* Abstract only. *ERA* Early RA, *EstRA* Established RA, *HC* Healthy control, *csDMARDs* Conventional synthetic disease odifying anti-rheumatic drugs, *bDMARDs* Biologic disease-modifying anti-rheumatic drugs, *TNFi* Tumour Necrosis Factor inhibitor drug, *ADA* Adalimumab, *ETN* Etanercept, *antiIL-6R* Anti-interleukin 6 receptor, *ELISA* Enzyme-linked immunosorbent assay, *ECLA* Electrochemiluminescent (ECLA), *MSD* Meso Scale Discovery, *qPCR* Quantitative polymerase chain reaction (qPCR), *DAS28* Disease activity score for 28 joint count, *SJC* Swollen joint count, *TJC* Tender joint count, *PGA* Patient global assessment, *RF* Rheumatoid factor, *ACPA* Anti–citrullinated peptide antibodies

### CXCL13 assays

CXCL13 was measured in serum in 25 studies, in synovial tissue in 4 studies, and in both serum and synovial tissue in the remaining 2 studies (Tables 1 and 2). The majority of studies quantified CXCL13 levels using commercial enzyme-linked immunosorbent assay (ELISA) kits, although an assortment of manufacturers was sourced. The remaining studies used Luminex -based assays [35], electrochemiluminescent (ECLA) [40], or a combination of ELISA, Luminex or Meso Scale Discovery (MSD) [18, 41]. Six studies did not mention the immunoassay method used [19, 30, 33, 34, 37, 42]. The method of measurement is of importance as data generated with Luminex technology is sensitive to heterophilic antibodies in the serum, such as rheumatoid factor and may result in false positive cytokine levels. Some studies analysed values as continuous variables whilst other studies employed a predefined cut off or a level above the median.

**Table 2:** Changes in CXCL13 levels with different therapies in RA

Study	Population	Study	Substrate	CXCL13 levels with treatment
Moura et al. (2017) [15]	*N* = 33. ERA, EstRA Anti-TNF, anti-IL6R	Cohort	Serum by ELISA	No change with: ADA, GOL, ETN or TCZ
Rinaldi et al.* (2017) [33]	*N* = 34. EstRA Anti-IL6R	RCT	Serum, method not published	Reduction with anti-IL-6 (TCZ, vobarilizumab)
Han et al. (2016) [16]	*N* = 29. EstRA Anti-TNF	Cohort	Serum by ELISA	Reduction with TNF (ADA/ETN) in TNF responders B/L high level associated greater response
Bugatti et al.* (2016) [34]	*N* = 205. ERA csDMARD	Cohort	Serum, method not published	B/L high levels predict failure of remission
Kumagai et al.* (2016) [17]	*N* = 146. EstRA Anti-TNF	Cohort	Serum by ELISA	Reduction with ADA in ADA responder. Not seen with IFX
Loza et al.* (2016) [18]	*N* = 916. ERA Anti-IL6	Cohort	Serum by ELISA, MSD, Luminex	No change with sirukumab
Bugatti et al.* (2016) [19]	*N* = 213. EstRA csDMARD	Cohort	Both, method not published	B/L high levels predict failure of remission
Greisen et al. (2014) [23]	*N* = 114. ERA csDMARD, Anti-TNF	Cohort	Serum by ELISA	Correlate with treatment response. B/L high level predict ADA treatment remission
Bugatti et al.* (2014) [24]	*N* = 205 csDMARD	Cohort	Serum by ELISA	No change with treatment
de Jong et al. (2014) [35]	*N* = 18 Anti-CD20	Cohort	Serum by Luminex	Reduction with rituximab irrespective of responder status
El-Sherbiny et al.* (2013) [36]	*N* = 23 Anti-CD20	Cohort	Synovial by mRNA qPCR	Reduction with rituximab in rituximab responders
Taylor et al. (2017) * [37]	*N* = 65 EstRA JAKi	RCT	Serum, method not published	Reduction with filgotinib. No change with placebo
Gabay et al. (2016) [38]	*N* = 356 EstRA Anti-IL6R	RCT	Serum by ELISA	Reduction with sarilumab (*p* < 0.01) from 2 weeks
Boyle et al. (2015) [39]	*N* = 29 EstRA JAKi	RCT	Serum by ELISA	Reduction with tofacitinib. No change with placebo
Dennis et al. (2014) [40]	*N* = 198. EstRA Anti-TNF, anti-IL6R	RCT	Serum by ECLA	Low B/L levels associate greater response to ADA High B/L levels associated greater response to TCZ
Kennedy et al. (2014) [41]	*N* = 214. EstRa Anti-TNF, antiLTα	RCT	Serum by ELISA & MSD	Reduction with ADA and pateclizumab.
Herman et al. (2013) [42]	*N* = 325. EstRa Anti-TNF, anti-IL6	RCT	Serum, method not published	Reduction with ADA, correlates with ADA response Reduction with TCZ, B/L high levels predict response
Emu et al. * (2012) [43]	*N* = 65 antiLTα	RCT	Serum by ELISA	Reduction with pateclizumab
Rosengren et al. (2011) [44]	*N* = 24, Anti-CD20	Cohort	Both by ELISA, mRNA qPCR	Reduction with rituximab at 2 months, which was maintained at 6 months

* Abstract only. *ERA* Early RA, *EstRA* Established RA, *HC* Healthy control, *csDMARDs* Conventional synthetic disease-modifying anti-rheumatic drugs, *bDMARDs* Biologic disease-modifying anti-rheumatic drugs, *TNFi* Tumour Necrosis Factor inhibitor drug, *ADA* Adalimumab, *ETN* Etanercept, *antiIL-6R* Anti-interleukin 6 receptor. *DAS28* Disease activity score for 28 joint count, *SJC* Swollen joint count, *TJC* Tender joint count, *PGA* Patient global assessment, *RF* Rheumatoid factor, *ACPA* Anti–citrullinated peptide antibodies

### Baseline levels of CXCL13 in patients with rheumatoid arthritis

Twelve studies reported baseline CXCL13 levels compared to healthy controls; 7 in early RA, 3 in established disease, and 2 studies analysed levels in both patient groups (Table 1). All of the studies reported higher levels of CXCL13 in RA compared to healthy controls. Moura et al. [15] compared levels in early RA to that seen in established disease and did not detect significant differences.

### CXCL13 as a marker of disease activity and severity

Sixteen studies evaluated how well levels of CXCL13 correlated with other measures of RA disease activity (Table 1). These included; tender joint counts (TJC), swollen joint counts (SJC), ESR, CRP and Disease Activity Score with ESR (DAS28-ESR) and CRP (DAS28-CRP) and ultrasound. Twelve of the sixteen studies reported a positive correlation between markers of disease activity and CXCL13. For the most part, these were weakly positive correlations, with rho values between 0.20–0.67. The strongest correlation was reported in association with DAS28-ESR [28]. Correlations were also noted with DAS28-CRP [17, 26, 27]. Six studies reported on the individual components of the DAS28 score. In half of these baseline CXCL13 weakly correlated with the swollen joint count with rho values between 0.23–0.34 [23, 31, 32]. The majority of studies used the 28-joint count. No significant correlation was observed with the tender joint count. One study reported a correlation with patient-derived measures (patient global assessment) [23]. Four studies examined CXCL13 as a marker of ultrasound synovitis, reporting a correlation with ultrasonographic scores for Grey Scale (*r* = 0.27) [24, 31] and Power Doppler signals (*r* = 0.26–0.69) [24, 21, 29, 31]. Four studies evaluated the association between CXCL13 level and seropositivity (RF and CCP). CXCL13 levels were found to be higher in RF and ACPA positive patients [16, 19, 32], with a positive correlation between synovial CXCL13 expression and ACPA titres [22]. Two studies assessed the association with CXCL13 and radiological evidence of erosive disease, demonstrating a strong association with progression of joint destruction in patients with high baseline CXCL13 levels [32].

### Baseline and change in CXCL13 levels with treatment

Nineteen studies evaluated changes in CXCL13 levels with treatment (Table 2). The therapies evaluated included conventional synthetic DMARDs (*n* = 4), TNF inhibitors including adalimumab, etanercept, infliximab and golimumab (*n* = 7), IL-6 cytokine and receptor blockers including tocilizumab, vobarilizumab, sarilumab (*n* = 6), anti-CD20 B cell depleting therapy rituximab (*n* = 2), JAK inhibitors tofacitinib and filgotinib (*n* = 2) and anti-lymphotoxin alpha agent pateclizumab (*n* = 2).

In csDMARD treated patients, high baseline levels of CXCL13 were associated with a reduced likelihood of achieving low disease activity. Bugatti reported a high negative predictive value of 80% for remission after 12 months of methotrexate therapy [19]. With anti-TNF therapy, the findings were not consistent across studies. Three studies reported reductions in CXCL13 levels with adalimumab and etanercept in treatment responders [16, 17, 42]. Kennedy et al. reported a significant reduction in CXCL13 with adalimumab compared to placebo, with levels returning to pre-dose values during the safety follow-up [41]. However, Moura et al. found no changes in CXCL13 levels before and after therapy although the authors did not stratify by therapeutic response [15]. The inconsistencies may be explained by Dennis et al. observation, that TNF response rates are influenced by the predominate RA synovial phenotype. High serum CXCL13 accompanied a lymphoid phenotype which associates with a lower response rate to TNF, compared to patients with a myeloid phenotype which associated with low CXC13 levels and high soluble intercellular adhesion molecule 1 levels [40]. Similar discrepancies were reported with anti-IL6 therapy. Rinaldi et al. reported a gradual reduction in CXCL13 levels with vobarilizumab and tocilizumab, with levels increasing back to pre-treatment levels on treatment discontinuation [33]. Gabay et al. reported a statistically significant reduction in CXCL13 levels with sarilumab, which was greatest in treatment responders [38], whilst Loza et al. did not document any change with sirukumab treatment [18].

Reduction in levels were also noted with rituximab, tofacitinib and filgotinib and pateclizumab. With rituximab, CXCL13 levels significantly decreased in all patients coinciding with B cell depletion irrespective of responder status [35, 36, 44]. Baseline levels did not predict response to therapy, however low levels predicted a lower B cell return at 6 months compared to high baseline level [44]. The JAK inhibitors (tofacitinib and filgotinib) reduced a range of RA associated tissue derived biomarker including CXCL13 [37, 39]. In the pateclizumab studies, CXCL13 were evaluated as a biomarker for target modulation. Levels decreased rapidly and significantly compared to treatment with placebo and returned to pre-dose levels during safety follow up. Despite reductions in CXCL13 levels, improvement in disease activity was not statistically significant [41, 43].

## Discussion

To our knowledge, this is the first systematic review exploring whether CXCL13 is a clinically viable diagnostic marker in RA. CXCL13 is raised in RA, both in early and established disease. Serum levels may predict active joint inflammation and synovitis over current biomarkers. Although levels fall in response to certain RA therapies, the prognostic value of CXCL13 in treatment response remains unclear.

CXCL13 is a marker of synovial inflammation in patients with RA. Levels are significantly higher than seen in healthy controls, present in very early RA (even in cases of less than 6 weeks disease duration) [25] and in both seropositive and seronegative disease, signifying a promising role as a diagnostic marker. However CXCL13 is also expressed in other inflammatory diseases driven by lymphoid organization, for example Sjogren’s syndrome, where levels correlate with the extent of salivary gland inflammation and subsequent lymphoma development [28].

Serum CXCL13 levels can be used as a surrogate marker for synovitis. CXCL13 is produced by multiple cell types within the synovium, including T cells, monocytes/macrophages, endothelial cells and fibroblast [25]. A number of synovial inflammatory markers correlate with CXCL13 synthesis [31, 44] and levels reflect the extent of synovial inflammation in clinical and imaging evaluation [31]. The inflamed rheumatoid synovium significantly contributes to serum CXCL13 levels, with a strong relationship between synovial expression and serum level [24, 26]. This is in contrast to acute phase proteins, which are primarily produced by hepatocytes released during inflammation, or other cytokines expressed in the synovium which are low or undetectable in the serum. In this study we found that CXCL13 levels correlated with swollen joint counts and ultrasound findings of synovitis. It is biologically plausible that serum CXCL13 levels directly reflect synovial production and synovial inflammation. Measurement of CXCL13 may help to predict active joint inflammation, yielding this biomarker a valuable surrogate in the objective assessment of the synovitis.

However, the evidence surrounding the correlation between CXCL13 and markers of disease activity is conflicting. Some studies report associations with DAS28, CRP, ESR and swollen joint count, whilst other studies have found no such correlation. There are several possible reasons for this. The DAS28 score does not solely reflect active disease driven by inflammation. The more subjective components of the score (tender joint count and patient global assessment) could be driven by non-inflammatory mediators, which may explain the lack of association with CXCL13. Unlike serum CXCL13 which is primarily produced by the inflamed synovial tissue, CRP is produced by hepatocytes in response to interleukins released during inflammation and may not directly reflect synovial inflammation. This may explain the absence of correlation between these two markers [31]. There is also disagreement regarding the association between CXCL13 and radiographic joint destruction. Associations with erosive disease were seen in patients with established RA [22, 32] but not in those with early disease [31]. This may relate to the homogeneousness in radiographic disease seen within the early RA cohort.

RA is a heterogeneous disease with a variety of clinical presentations and differences in responsiveness to treatment modalities. Considerable molecular heterogeneity has been reported and different patient subgroups proposed on the basis of gene expression patterns and predominant synovial cell infiltrates [45]. The lymphoid phenotype had a high expression of B cell-associated genes and synovial CXCL13 levels whilst the myeloid phenotype demonstrates higher levels of other important synovial cytokines such as TNFα [40]. This may explain the conflicting evidence regarding the association with CXCL13 and disease activity, especially as studies have not defined their populations by predominant synovial infiltrates. In studies that did examine cellular infiltrates, synovial CXCL13 expression associated with an active lymphoid cell and a more severe erosive disease pattern [22].

Multiple studies reported that CXCL13 levels reduced in response to treatment with biologics including anti-TNF, anti-IL6, B cell depletion and small molecule JAK inhibitor. In some studies, the reduction in CXCL13 was only seen in treatment responders. These finding were not duplicated in all studies. Three studies found no change in levels with treatment. With methotrexate initiation, in contrast to the early reduction in acute phase markers, CXCL13 levels did not significantly change, reflecting a more persistent pattern of synovial inflammation [24].

As a predictor of treatment response, evidence is also conflicting. In patients receiving csDMARDs, CXCL13 levels predicted failure for disease remission, especially when combined with autoantibody positivity [19, 34]. With anti-TNF therapy, two studies reported high baseline levels associated with greater treatment response [16, 23]. In contrast, Dennis et al. reported low baseline levels associated with anti-TNF treatment response, whilst high baseline levels associated with a greater response to tocilizumab [40], a finding duplicated in an earlier study [42]. A biologically plausible mechanism was suggested to be linked to the predominant synovial phenotype. Patients with low baseline CXCL13 (and high soluble intercellular adhesion molecule) were more likely to have a myeloid inflammatory phenotype, with an overrepresentation of TNFα-associated gene expression, and hence a better response to TNFα blockade [40]. When considering these results, it is important to note variation in measurements of CXCL13 between studies, with some employing a predefined cut off of > 100 pg/ml, and others adopting a level above the median, or even a ratio computed with sICAM1 levels. It is conceivable that different assays are confounders.

There are limitations to this review. The most significant of these is the heterogeneity of the research questions and results between the studies reviewed. Each employed a different study design, with diverse study populations and analyses of an assortment RA therapies. The samples size across the studies ranged from 18 to 1135. Without a meta-analytical approach, it is impossible to incorporate the impact from a large study into the results. Disease activity, severity and treatment response were measured by a variety of different outcomes, limiting the ability to make direct comparisons.

A further limitation relates to the variability in CXCL13 assays. It is important to recognise that CXCL13 assays are primarily used in the research setting and are not accredited clinical laboratory assays. There is significant potential variation across technologies (ELISA, Luminex etc) and laboratories. We have presented the differences in methodology across the studies to make sure this aspect of variation is fully appreciated. The timing of biomarker collection, e.g. at time of assessment or at disease flare, was also not matched across the studies. In addition, as mentioned earlier, a number of studies used binary thresholds for CXCL13 rather than presenting absolute values which needs to be considered when interpreting the results presented.

## Conclusion

In summary, CXCL13 expression is raised in a subset of patients with early and established RA, and levels may fall in response to therapy. It may have a discrete role as a biomarker in selected synovial pathotypes as a surrogate indicator of lymphoid organogenesis and active joint inflammation. In our opinion, there is not yet evidence for CXCL13 to predict disease progression or treatment response when used alone, partly because of the heterogeneity of the research questions and results from the studies reviewed here. We conclude that at present, the results are insufficient to justify the routine use of CXCL13 as a biomarker in the clinic setting. More research is required. For example, a study designed to recruit patients with new onset inflammatory arthritis, defined phenotypically and stratified to receive therapy based on serum CXCL13 levels would be of considerable interest. Without such a trial to test the hypothesis that CXCL13 is a useful biomarker, it is difficult to make a firm recommendation regarding its use in RA.

## Supplementary information

**Additional file 1.**

## Data Availability

All data generated or analysed during this study are included in this published article.

## Abbreviations

RA: Rheumatoid arthritis
ERA: Early RA
EstRA: Established RA
HC: Healthy control
csDMARDs: Conventional synthetic disease modifying anti-rheumatic drugs
bDMARDs: Biologic disease-modifying anti-rheumatic drugs
TNFi: Tumour Necrosis Factor inhibitor drug
ADA: Adalimumab
ETN: Etanercept
antiIL-6R: Anti-interleukin 6 receptor
ELISA: Enzyme-linked immunosorbent assay
ECLA: Electrochemiluminescent (ECLA)
MSD: Meso Scale Discovery
qPCR: Quantitative polymerase chain reaction (qPCR)
DAS28: Disease activity score for 28 joint count
SJC: Swollen joint count
TJC: Tender joint count
PGA: Patient global assessment
RF: Rheumatoid factor
ACPA: Anti–citrullinated peptide antibodies
