# Supplementary material for: A systematic review of CXCL13 as a biomarker of disease and treatment response in rheumatoid arthritis
Source: BMC Rheumatol. 2020 Nov 2;4:70. doi: 10.1186/s41927-020-00154-3 (PMC7604968; doi:10.1186/s41927-020-00154-3)
Supplement: Supplementary file 1 — Additional file 1. [file 41927_2020_154_MOESM1_ESM.docx]

Supplementary figure 1: Cochrane risk of bias assessment for RCT

|  | Random sequence generation  (selection bias) | Allocation concealment  (selection bias) | Blinding participant & personal (performance bias) | Blinding outcome assessment  (detection bias) | Incomplete outcome date  (attrition bias) | Selective reporting  (reporting bias) |
| --- | --- | --- | --- | --- | --- | --- |
| **RCTs** | | | | | | |
| Greisen et al 2014 (12) | Patients were randomized in blocks of four from a central, computer-generated list of study numbers. | Not described | Adalimumab replaced with placebo-adalimumab | Not described | Attrition and exclusion was reported on. How many discontinued and reasons given. | Efforts made to ensure the same doctor followed each patient. Standardised scoring systems used at each meeting (e.g. VAS and number of swollen and tender joints). Serum levels chemokines taken. |
| Herman et al (ADACTA) 2013 (20)- Abstract | Not described | Not described | Patients either given ADA+placebo TCZ or TCZ+ placebo ADA | Not described | Not described | Standardised scoreing systems used at patient visits. (e.g. DAS28, ACR 20/50/70, HAQ DI). Serum levels chemokines taken. |
| Kennedy et al 2014 (ALTARA) (22) | Method for random sequence generation not described. | Not described | Medical professional administering the ADA unblinded as the manufacturer and placebo syringe were different. Strict blind for patients was maintained. Possibility for bias as the syringes were not corresponding | Strict blind for outcome assessors was maintained | Attrition and exclusion was reported on. How many discontinued and reasons given. | Standardised scoreing systems used at patient visits (e.g. DAS28, ACR 20/50/70, HAQ DI). Missing ACR scores were inputted with the last observation carried forward method. Serum levels chemokines taken. |
| Rinaldi et al 2017 (24)-Abstract | Not described | Not described | Not described | Not described | Not described | Not described |
| Gabay et al 2016 (TARGET) (25)- Abstract | Randomization performed centrally | Not described | Not described | Independent assessor of joints, with no access to patient data, performed SJC and TJC measurements. Investigators were blinded with regard to the patients’ CRP level, serum sarilumab levels, and anti−sarilumab antibody positivity, except at screening and baseline | Patient rescued by Sarilumab at 12 weeks excluded from the analysis. | Standardised scoreing systems used at patient visits (e.g. DAS28, ACR 20/50/70, HAQ DI). Serum levels chemokines taken. |
| Taylor et al 2017 (DARWIN-2) (30) Abstract | Patients randomly assigned to treatment using a computerised interactive voice and web response system. | Not described | Not described | Not described | Attrition and exclusion was reported on. How many discontinued and reasons given. | Standardised scoreing systems used at patient visits (e.g DAS28, ACR 20/50/70, HAQ DI). Serum levels chemokines taken. |
| Boyle et al 2015 (31) | Not described | Not described | Not described | Not described | No patients left the trial | Standardised scoreing systems used at patient visits (e.g DAS28, ACR 20/50/70). Serum levels chemokines taken. |
| Emu et al 2012 (32) | Not described | Not described | Not described | Not described | Attrition and exclusion was reported on. How many discontinued and reasons given. | Standardised scoreing systems used at patient visits (e.g DAS28, ACR 20/50/70). Serum levels chemokines taken. |
| Sellam et al 2013 (37) | Not described | Not described | Open label | Open label | Not described | Standardised scoreing systems used at patient visits (e.g EULAR). Serum levels chemokines taken. |

Supplementary figure 2: Newcastle – Ottawa Quality assessment scale

| Case control studies | Selection  1. case definition  2. representativeness of cases  3. selection of controls  4. definition of controls | Comparability | Exposure   1. Ascertainment 2. Same for cases & controls 3. Non-Response rate | NOS Score |
| --- | --- | --- | --- | --- |
| Pandya et al 2017 (33) | ******* | ****** | ****** | **7** |
| Moura et al 2017 (10) | ******* |  | ******* | **6** |
| Loza et al 2016 (26) | ***** | ***** | ****** | **4** |
| Han et al 2015 (34) | ******** | ****** | ****** | **8** |
| Moura et al 2014 (36)- abstract | ****** |  | ****** | **4** |
| Sherif et al 2013 (11) | ******* | ****** | ****** | **6** |
| Setiadi et al 2013 (38) |  |  | ****** | **2** |
| Ahmed et al 2013 [16] | ******* | ***** | ****** | **6** |
| Cohort Studies | Selection  1. representativeness of exposed  2. selection of non-exposed  3. ascertainment of exposure  4. interest not present at start | Comparability | Outcome   1. Assessment of outcome 2. Long enough follow up 3. Adequacy o follow up 4. Same for cases & controls 5. Non-Response rate | NOS Score |
| Han et al 2016 (18) | ******** |  | ****** | **6** |
| Kumagi et al 2016 (21)- abstract | ***** |  | ****** | **3** |
| Bugatti et al 2016 (19) - abstract |  |  | ***** | **1** |
| Bugatti et al 2014 (15) - abstract | ***** |  |  | **1** |
| De Jong et al 2014 (27) - abstract | ***** |  |  | **1** |
| El-Sherbiney at el 2013 (28)- abstract | ***** |  |  | **1** |
| Jones et al 2014 (41) | ******* | ****** | ***** | **6** |
| Bugatti et al 2012 (13) | ******* | ***** | ******* | **8** |
| Meeuwisse et al 2011 (14) | ******* |  | ******* | **6** |
| Bugatti et al 2012 (39) | ******* | ****** | ****** | **6** |
| Dennis et al 2014 (23) | ****** |  | ****** | **4** |
| Rosengren et al 2011 (29) | ******* |  | ***** | **4** |

Additional studies

- Both Bugatti et al 2014 [35] and Yeo et al 2015 [17] were cross sectional studies.
